# Supplementary material for: Is Hypnotic Induction Necessary to Experience Hypnosis and Responsible for Changes in Brain Activity?
Source: Brain Sci. 2023 May 29;13(6):875. doi: 10.3390/brainsci13060875 (PMC10296668; doi:10.3390/brainsci13060875)
Supplement: Supplementary file 1 [file brainsci-13-00875-s001.zip › brainsci-2384581-Figures S1-S3.pdf]

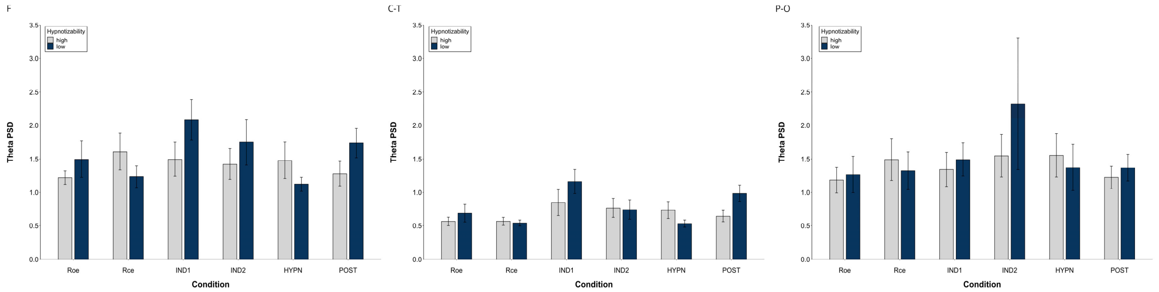

Figure S1. Theta PSD in the frontal, central-temporal and parieto-occipital regions during each experimental condition (ROE, RCE, IND1, IND2, NH, Post). Side and regions averaged.

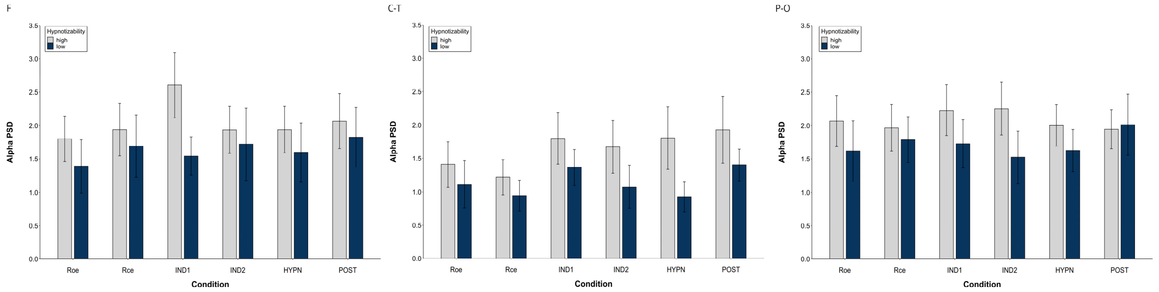

Figure S2. Al-pha PSD in the frontal, central-temporal and parieto-occipital regions during each experimental condition (ROE, RCE, IND1, IND2, NH, Post). Side and regions averaged.

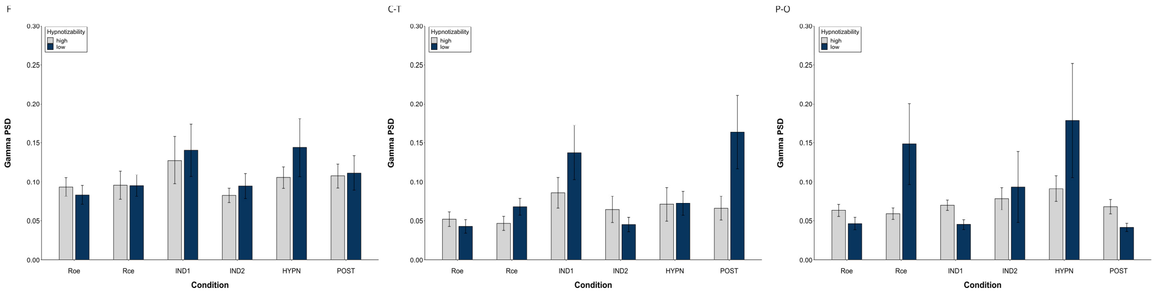

Figure S3. Gamma PSD in the frontal, central-temporal and parieto-occipital regions during each experimental condition (ROE, RCE, IND1, IND2, NH, Post). Side and regions averaged.
